# Vancomycin Prescribing Practices and Therapeutic Drug Monitoring for Critically Ill Neonatal and Pediatric Patients: A Survey of Physicians and Pharmacists in Hong Kong

**DOI:** 10.3389/fped.2020.538298

**Published:** 2020-11-30

**Authors:** Twinny Cheuk Hin Chow, Janice Yuen Shun Li, Jasper Chak Ling Wong, Freddie Man Hong Poon, Hugh Simon Lam, Teddy Tai-ning Lam, Chui Ping Lee, Celeste Lom-ying Ewig, Yin Ting Cheung

**Affiliations:** ^1^Department of Pharmacy, Hong Kong Children's Hospital, Hong Kong, China; ^2^Department of Paediatrics, Faculty of Medicine, The Chinese University of Hong Kong, Hong Kong, China; ^3^School of Pharmacy, Faculty of Medicine, The Chinese University of Hong Kong, Hong Kong, China; ^4^Department of Pharmacy, Prince of Wales Hospital, Hong Kong, China

**Keywords:** critically ill, therapeutic monitoring of antibiotic levels, pediatric ICU, neonatal ICU, vancomycin

## Abstract

**Background:** Deviations from the optimal vancomycin dosing may occur in the neonatal and pediatric population due to inconsistencies in the recommended dosing algorithms. This study aims to collect the expert opinions of clinicians who practice in the neonatal or pediatric intensive care units (NICU/PICUs) of 12 major medical centers in Hong Kong.

**Methods:** This was a multicenter, cross-sectional study. Eligible physicians and pharmacists completed a structured questionnaire to identify the challenges they encountered when selecting the initial intermittent vancomycin dosing. They also answered questions concerning therapeutic monitoring services (TDM) for vancomycin, including the targeted trough levels for empirical vancomycin regimens administered for complicated and uncomplicated infections.

**Results:** A total of 23 physicians and 43 pharmacists completed the survey. The top clinical parameters reported as most important for determining the initial vancomycin dosing were renal function (90.9%), post-menstrual/postnatal age (81.8%), body weight (66.7%), and suspected/documented pathogen (53.0%). Respondents reported challenges such as difficulties in determining the optimal initial dose for a targeted level (53.0%), inconsistencies between dosing references (43.9%) and a lack of clear hospital guidelines (27.3%). Half of the pharmacists (48.8%) reported that they had helped to interpret the TDM results and recommend vancomycin dose adjustments in >75% of cases. For methicillin-resistant *Staphylococcus aureus* infection, physicians, and pharmacists reported target trough levels of ~10–15 and 15–20 mg/L, respectively. For suspected moderate/uncomplicated Gram-positive infections physicians tended to prefer a lower trough range of 5–10 mg/L, while pharmacists preferred a range of 10–15 mg/L.

**Conclusions:** Our results demonstrate that clinicians used varying vancomycin dosing guidelines in their practices. The multidisciplinary TDM service in Hong Kong can be improved further by establishing a standardized dosing guideline and implementing a well-structured, evidence-based service protocol. Future work includes conducting drug utilization studies to evaluate real-world antimicrobial usage patterns and the impact on tangible clinical outcomes, and developing pharmacokinetic-guided dose calculator for antimicrobials in critically ill neonates and pediatric patients.

## Key Points

- Clinicians used varying dosing guidelines in their selection of initial IV vancomycin dosing for patients of NICU/PICUs in Hong Kong.
- Despite the purported benefits of therapeutic drug monitoring (TDM) services, the availability and scope of this clinical service varies among local public hospitals.
- A standardized dosing guideline is needed to decrease variability and improve local practice.
- Future work includes conducting drug utilization studies and developing pharmacokinetic-guided dose calculator for antimicrobials in critically ill neonates and pediatric patients.

## Introduction

Critically ill neonatal and pediatric patients face a higher risk of nosocomial infection due to a combination of immature humoral and cellular immunity and the use of invasive techniques such as central venous catheterization and endotracheal intubation. Neonatal bacterial sepsis causes death in 10–20% of cases and morbidity in 25–30% of survivors. Therefore, effective and timely antimicrobial treatment regimens are often life-saving (1).

Selection of antimicrobial therapy for a critically ill patient may vary based on the nature of treatment (empirical or directed), source of infection and/or likely pathogen(s) and susceptibility. Vancomycin, a glycopeptide antibiotic, is often prescribed to cover resistant Gram-positive organisms such as methicillin-resistant *Staphylococcus aureus* (MRSA) and coagulase-negative *Staphylococci* (CoNS), particularly methicillin-resistant coagulase-negative *Staphylococci* (MRCoNS). MRSA is among the most threatening pathogens and the principal indicator of nosocomial risk. The incidence of neonatal sepsis has also increased over time in developed countries among very low-birthweight (VLBW) infants, and CoNS has emerged as a major pathogen in this context (2, 3). In a NICU/PICU setting, empirical vancomycin therapy is commonly initiated in a patient with suspected staphylococcal infections until the susceptibility pattern of the pathogen is confirmed.

The optimization of the initial vancomycin dosing required to achieve a therapeutic plasma level, and reducing the time taken to reach this level, have been proven to improve clinical outcomes in adults (4). However, deviations from the optimal vancomycin dosing may occur in the neonatal and pediatric population due to inconsistencies in the recommended dosing algorithms (5–8). This lack of consensus is illustrated by the wide variation in dosing regimens recommended in reference guidelines and the targeted therapeutic levels (Supplementary Materials 1, 2) (9–14). One study conducted at a pediatric tertiary care medical center in the United States observed that 35% of initial vancomycin courses were inappropriate (15). Another study observed that 74% of vancomycin dosing regimens administered according to the British National Formulary for Children (BNFc) recommendations did not achieve the target trough level (16). This issue is concerning because the inappropriate or suboptimal use of vancomycin can lead to treatment failure and enable the emergence of antibiotic resistance. Many of the above recommendations are supported by a relatively weak level of evidence, and the optimal dosing and monitoring of vancomycin remain controversial. Recent guidelines for vancomycin dosing suggest an area under the curve (AUC)/minimum inhibitory concentration (MIC) ratio >400 as the primary targeted therapeutic parameter to treat MRSA infections (17). The considerable variability in dosing recommendations of vancomycin is an ongoing challenge.

Addressing antibiotic resistance has been a global health agenda over the recent years, and is currently one of the top research priorities in Hong Kong. In 2019, a multidisciplinary team consisting neonatologist, pediatricians, and clinical pharmacists initiated a mixed-methods approach to address appropriate antimicrobial use in the pediatric population. The overarching aim is to implement personalized, pharmacokinetic-guided dosing of antimicrobials in critically ill patients in the NICU/PICU through a mixed-methods research approach (Supplementary Material 3). At the preliminary stage, this study was developed to collect the expert opinions of a representative sample of physicians and pharmacists who practice in the NICU/PICUs of 12 major medical institutions in Hong Kong. This survey aimed to describe the initial vancomycin dose selection practices used in these NICU/PICUs while focusing on the key factors influencing dose selection, preferred references, and target trough level. We also aimed to identify the challenges associated with initial vancomycin dosing and the corresponding therapeutic drug monitoring (TDM) practices.

## Methods

This multicenter cross-sectional survey was conducted between January and December 2018. Approval was obtained from The Chinese University of Hong Kong (CUHK) Survey and Behavioral Research Ethics Committee prior to study initiation.

### Respondents

Physicians and pharmacists were recruited from 12 major medical centers within the Hospital Authority (HA; Supplementary Material 4), the statutory body that manages all public hospitals in Hong Kong, using a convenience sampling approach. Eligible respondents met the following criteria: (1) status as a practicing physician or pharmacist; (2) active practice in a participating NICU or/and PICU or general pediatric ward; and (3) prescribing of vancomycin (for physicians) or processing or dispensation of vancomycin prescriptions (for pharmacists) for at least one NICU or PICU patients per month. These criteria ensured that we targeted a homogenous sample of practitioners who were directly involved in the management of vancomycin for critically ill pediatric or/and neonatal patients.

### Survey Design

The investigators designed the survey based on the existing literature (18–21). The questionnaire comprised three sections (Table 1). The first section collected information about the respondents' clinical experiences, including their primary area(s) of practice and duration of healthcare practice (years). The second section comprised six questions designed to identify the references and clinical factors that respondents considered relevant when selecting the initial vancomycin dosing, as well as the encountered challenges. The third section comprised questions concerning TDM for vancomycin for different scenarios based on the type of infection and pathogens. This is because clinicians typically attempt to predict and narrow down the likely diagnosis and pathogenic bacteria based on the patient's signs/symptoms and clinical assessments when deciding empirical treatment. They would then make modifications to the treatment plan when the culture result is completed. The pre-identified scenarios include the targeted trough levels for empirical vancomycin regimens administered to a case of suspected uncomplicated and complicated Gram-positive infection and to a confirmed case of MRSA or CoNS infection. Examples of complicated infections included bacteremia, osteomyelitis, meningitis, severe pneumonia, endocarditis, and deep-seated infections. Intermittent infusion of vancomycin is assumed for all scenarios as continuous infusion is rarely administered in Hong Kong. Respondents were also asked to identify the reasons for not adjusting a patient's vancomycin dosing regimen in the event of a suboptimal trough level and the challenges of the current TDM services in their respective institutions. All questions were closed-ended, and the respondents were presented with choices as outlined in Table 1. A space for open-ended responses was provided at the end of the questionnaire for respondents to provide additional comments on any of the questions in the survey. The self-administered English-language survey required ~10 min for completion. The final versions of the surveys were revised through a pilot-test among seven physicians and pharmacists.

**Table 1:** Objectives, questions, and available options outlined in the questionnaire.

	**Objectives**	**Questions**	**Options**
1	To gather from practitioners their approach to vancomycin initial dosing regimen in neonatal and pediatric critical care patients	Which factor(s) is/are the most important consideration(s) in the selection of initial vancomycin dosing?	Patient-related: • Age (PMA/PNA for neonates) • Biological sex • Body weight • Renal function • Liver function • Fluid restriction/hydration status • Infection-related: • Target trough level • Site of infection • Suspected/documented pathogen • Severity of infection • Drug-related: • Concurrent nephrotoxic medications • Concurrent antibiotics
		Which drug reference(s) do you most often refer to in assessing the initial vancomycin dosing regimen in: • NICU patients? • PICU patients?	• UpToDate®/Lexicomp® Pediatric and Neonatal • Micromedex® Neofax and Pediatric Essentials • Frank Shann Drug Doses • BNF (British National Formulary) for Children • Local hospital guidelines • Disease-based guidelines • My own clinical judgement
2	To gather from practitioners their vancomycin TDM practices in neonatal and pediatric critical care patients	What is the approximate percentage of cases where TDM is ordered for: • NICU patients prescribed with vancomycin? • PICU patients prescribed with vancomycin?	• Never order • ~20% • ~40% • ~60% • ~80% • 100%
		For which reason(s) would you think Vancomycin does NOT require TDM?	• For empiric use pending culture results • For short-term use (<72 h) • For surgical prophylaxis • Prescribed dose always achieves target levels • Patient has good/normal renal function • Time lag for serum tests to be available • Target levels are unclear for unknown pathogen • Serum levels are not useful in patient management • Patient is hemodynamically unstable • TDM requires too much blood drawing • The patient does not have a severe infection
3	To gather from practitioners their recommended target trough ranges for common clinical scenarios at the PICU/NICU.	What is the target trough level (in mg/L) of an empirical vancomycin regimen (intermittent dosing) for: • Suspected moderate/uncomplicated Gram positive infections? • Suspected severe/complicated Gram positive infections? What would be the target trough level (for vancomycin intermittent dosing) if the patient's culture results come back indicating: • MRSA? • Methicillin-resistant CoNS?	For each scenario, respondents are asked to indicate the minimum and maximum targeted trough on a visual analog scale ranging from 1 to 20. Respondents are also given the option to state the target range for other vancomycin therapeutic parameters (e.g., AUC/MIC).
4	To gather from practitioners their perceived challenges with prescribing vancomycin/handling vancomycin prescriptions and TDM.	What challenges do you identify concerning empirical vancomycin dosing?	• Inconsistences among different dosing references • Lack of clear hospital guidelines • Difficult to determine dose for target serum level • Uncertainty of target serum level • Inadequate training in neonatal/pediatric antibiotic dosing • Lack of collaboration among healthcare professionals • No challenges identified
		In general, are you satisfied with the current system and practice of TDM in your institution?	• Very Satisfied • Satisfied • Neutral • Dissatisfied • Very dissatisfied
		What is/are limitations and challenges of the current TDM service in your institution?	Resource-related: • Long turnaround time for serum levels • Insufficient time to assess serum levels • No local guidelines • This service is not a key focus of the department Healthcare practitioner-related: • No cooperation/lack of support from physicians • Inadequate knowledge to know how to adjust the dosing regimen accordingly

### Statistical Analysis

We did not conduct a powered sample size calculation because of the descriptive nature of this study. Furthermore, we expected the sample size to be limited by the small cohort of healthcare professionals who specialize in pediatric and neonatal critical care medicine in Hong Kong. To avoid under representation of smaller institutions, we distributed a standard number of five copies of the survey to each institution to solicit their participation, as it is reasonable to assume that each institution should have at least five physicians and pharmacists providing service to the PICU/NICU. Department managers were asked to make additional copies when needed and track the distribution of the surveys.

Descriptive statistics were used to analyze the practitioners' responses to each question. Heat maps were used to depict graphically the different ranges of targeted trough values reported by the respondents. An exploratory analysis based on the Kolmogorov–Smirnov-test was conducted to examine differences in the distribution patterns of the targeted trough level ranges reported by physicians and pharmacists. All analyses were conducted using the Statistics Package for Social Science, version 25 (IBM, Armonk, NY: IBM Corp.).

## Results

### Overview

A total of 23 physicians and 43 pharmacists completed the survey, yielding response rates of 67.6 and 61.4%, respectively. The respondents' demographics are summarized in Table 2. Respondents provided service in 12 medical institutions which are distributed across the three main districts in Hong Kong (Supplementary Material 5). The majority of physicians (78.3%) and pharmacists (67.4%) indicated the NICU and/or PICU as their primary area of practice. More than 50% of physicians had more than 10 years of clinical experience, while most pharmacists had 1–7 years of experience in pediatrics. The respondents reported that they handled a median of 1–5 vancomycin cases per month in the NICU/PICU.

**Table 2:** Respondents' characteristics.

	**Physician (%) (*n* = 23)**	**Pharmacist (%) (*n* = 43)**
**Primary area of practice**		
NICU	4 (17.4)	3 (7.0)
PICU	3 (13.0)	20 (46.5)
Both NICU/PICU	11 (47.8)	6 (14.0)
General pediatrics	5 (21.7)	14 (32.6)
**Years of clinical experience**		
<1 year	1 (4.3)	3 (7.0)
1–3 years	4 (17.4)	21 (48.8)
4–7 years	4 (17.4)	15 (34.9)
8–10 years	2 (8.7)	3 (7.0)
>10 years	12 (52.2)	1 (2.3)
**Prescribe/encounter vancomycin cases in NICU**		
<1 per month	6 (26.1)	8 (18.6)
1–5 per month	10 (43.5)	20 (46.5)
5–10 per month	4 (17.4)	5 (11.6)
>10 per month	3 (13.0)	3 (7.0)
Do not encounter	0	7 (16.3)^*^
**Prescribe/encounter vancomycin cases in PICU**		
<1 per month	9 (39.1)	5 (11.6)
1–5 per month	8 (34.8)	25 (58.1)
5–10 per month	4 (17.4)	6 (14.0)
>10 per month	1 (4.3)	2 (4.7)
Do not encounter	1 (4.3)^*^	5 (11.6)^*^

* *Respondents indicated encounters with vancomycin cases in either PICU or NICU but not both*.

### Vancomycin Initial Dosing Regimen

Regarding the drug references used to assess vancomycin dosing in the NICU (Supplementary Material 6), ~60% of the respondents selected the Micromedex Neofax®, while 37.8 and 33.3% selected the UpToDate®/Lexicomp® and local hospital guidelines, respectively. Among respondents in the PICU, 59% selected the UpToDate®/Lexicomp®, while 39.4 and 33.3% selected the British National Formulary for Children (BNFc) and Drug Doses (Frank Shann), respectively (Supplementary Material 6).

**Figure 1:**
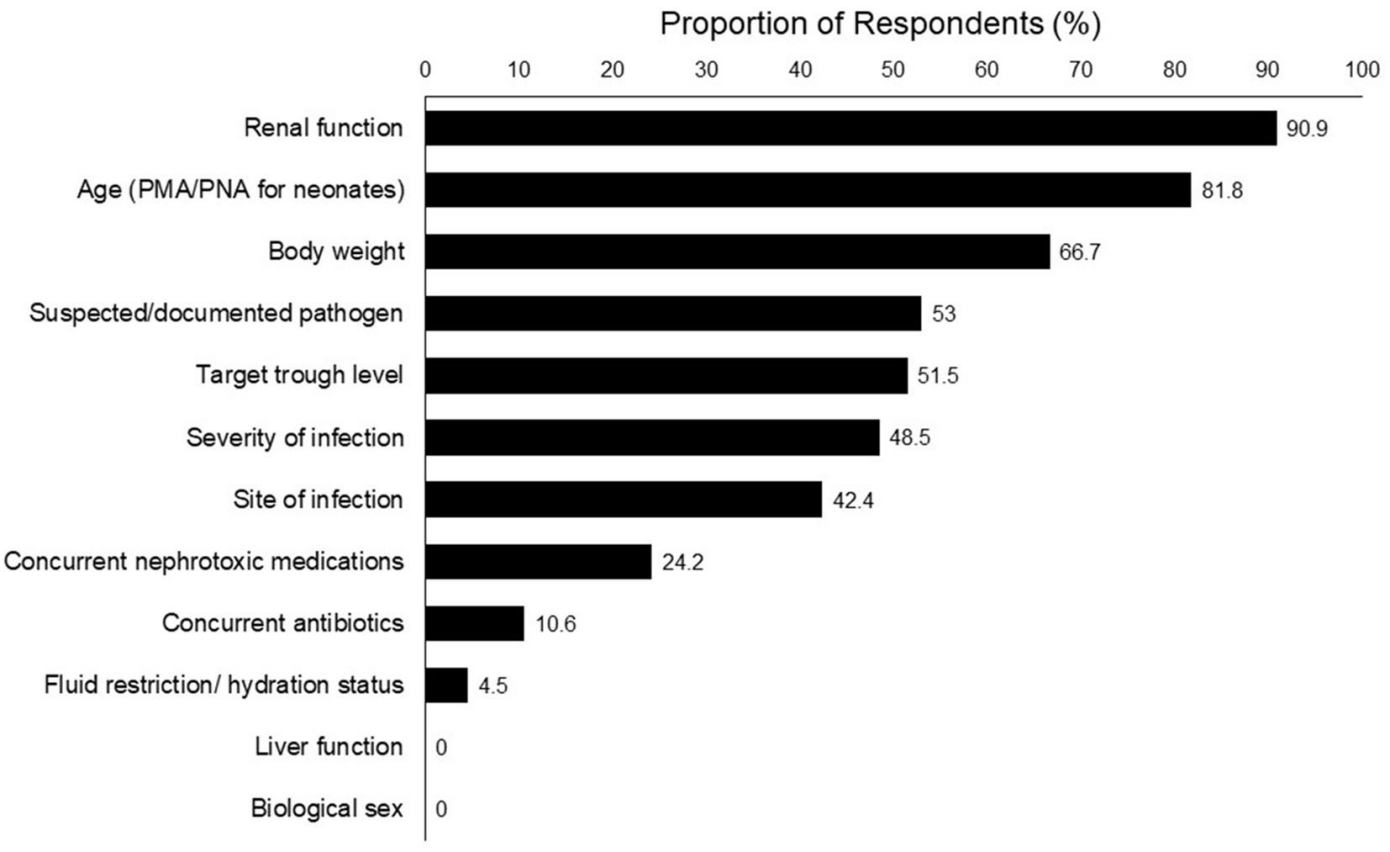
Top factors to consider in the selection of initial vancomycin dosing (*n* = 66).

The top 5 clinical parameters reported as most important for determining the initial vancomycin dosing were renal function (90.9%), age [post-menstrual age (PMA)/postnatal age (PNA) for neonates] (81.8%), body weight (66.7%), suspected/documented pathogen (53.0%), and target trough level (51.5%) (Figure 1).

A number of respondents provided open-ended responses on the rationale behind their dose selection. They expressed confusion regarding precise dosing due to varying dosing recommendations. One physician mentioned that in his/her opinion, the renal function is the most important factor to consider especially for a hemodynamically unstable patient, as it would directly affect the clearance of vancomycin. Pharmacists also indicated that they would consider the pharmacokinetics and pharmacodynamics profile of the concurrent medications because most critically ill patients are on multiple drugs, of which some may have a narrow therapeutic range.

### Challenges Concerning Empirical Dosing

**Figure 2:**
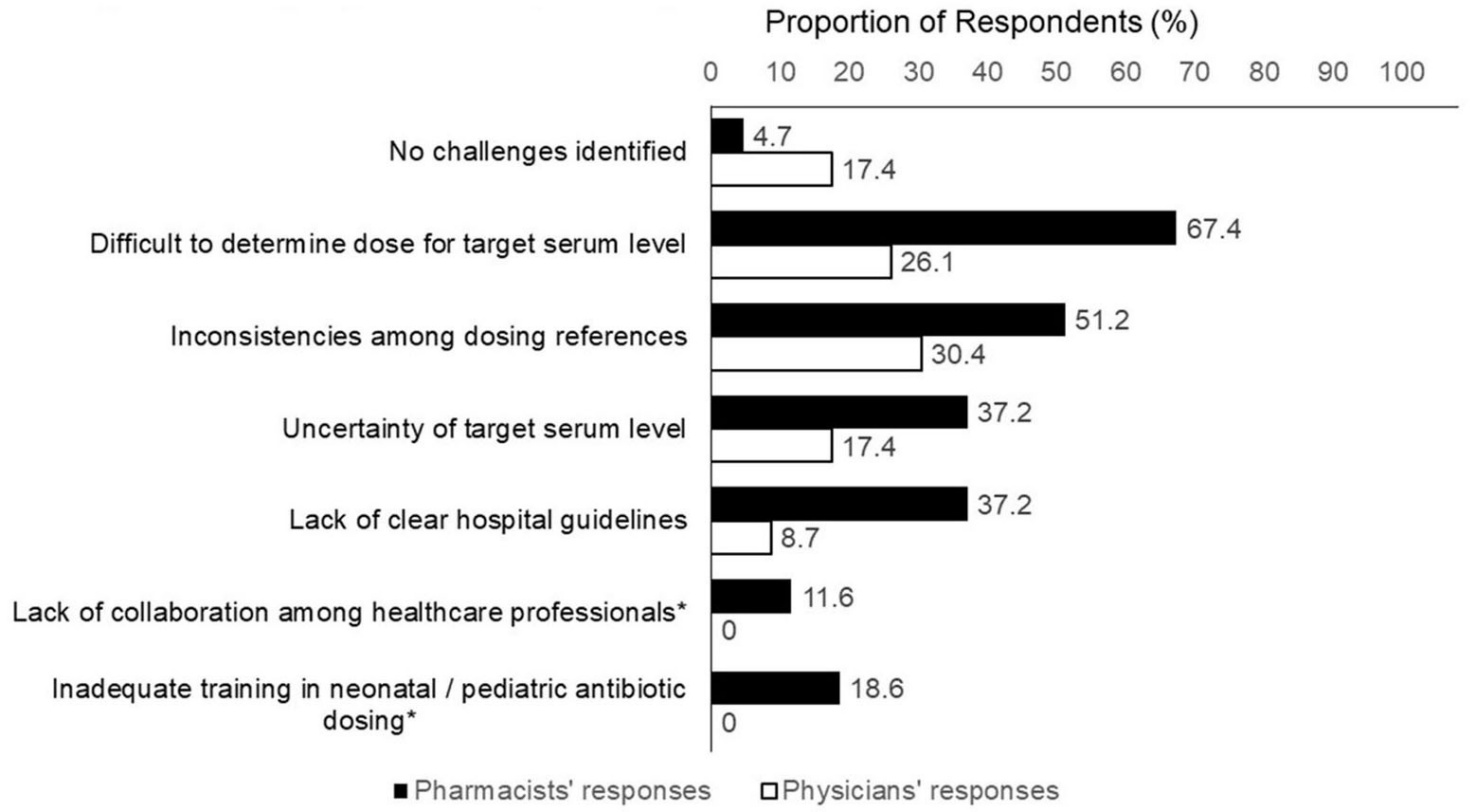
Challenges concerning empirical vancomycin dosing. *Item was not included in physician's version of questionnaire.

The respondents identified several challenges regarding the empirical vancomycin dosing (Figure 2). Physicians' challenges included uncertainty regarding the target serum level (37.2%), inconsistencies among different dosing references (30.4%), difficulty in determining the optimal initial dose for a targeted level (26.1%). There seemed to be a higher proportion of pharmacists who identified challenges with empirical dosing. More than two-thirds of the pharmacists (67.4%) considered it difficult to determine the optimal initial dose required to achieve a target serum level. Other challenges encountered by the pharmacists included inconsistencies between dosing references (51.2%) and a lack of clear hospital guidelines (37.2%).

### Vancomycin Therapeutic Drug Monitoring

The majority of respondents indicated that TDM was ordered for all vancomycin cases in the NICU (80.3%) and PICU (69.7%), and these respondents were from half of the represented institutions. Vancomycin TDM was not performed in certain situations where the drug was prescribed for surgical prophylaxis (65.2%), for short-term use (<72 h; 57.6%), and for empirical use while culture results are pending (18.2%) (Supplementary Material 7).

**Figure 3:**
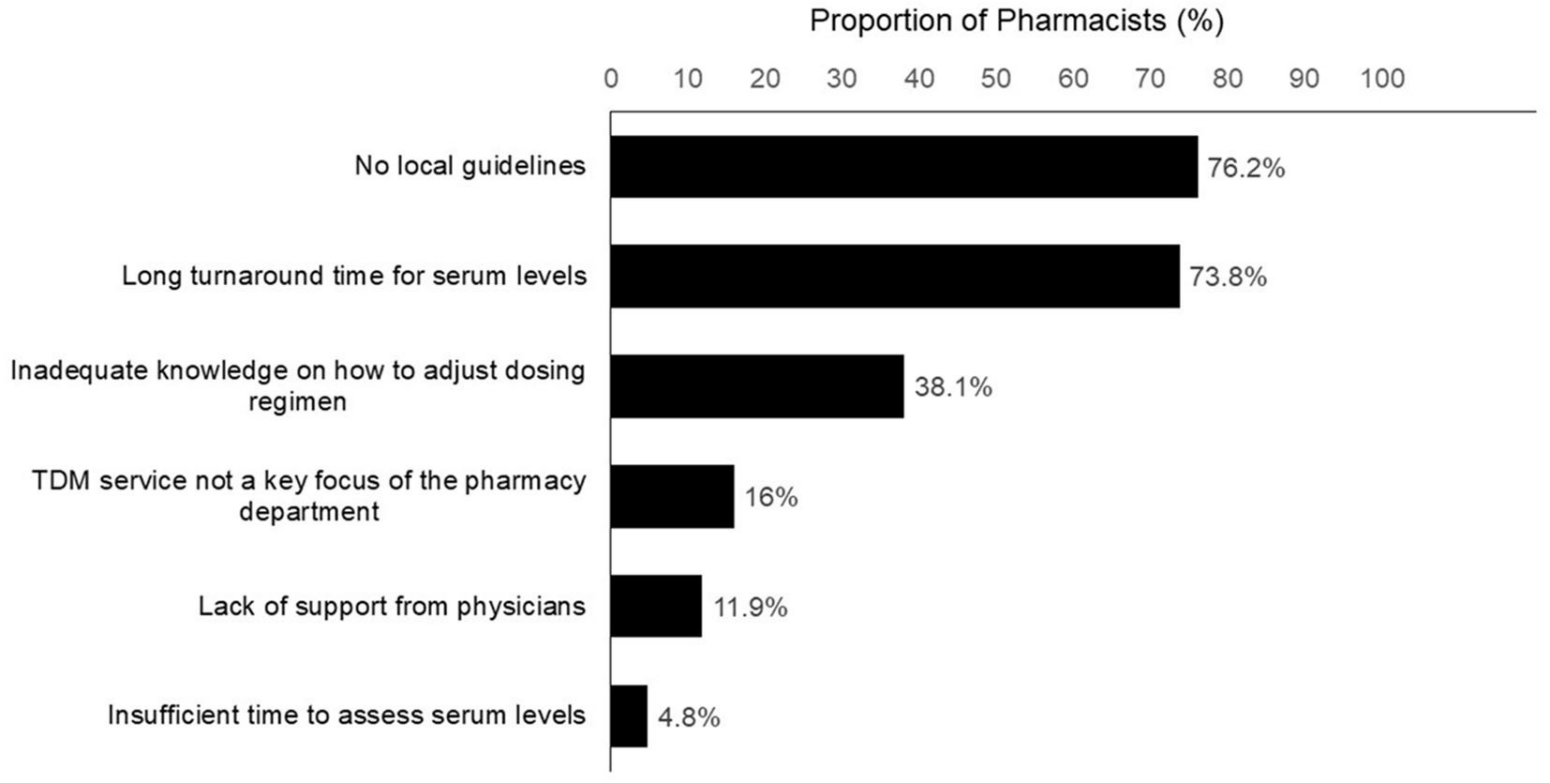
Challenges faced by pharmacists in establishing therapeutic drug monitoring service (*n* = 43).

Half of the pharmacists (48.8%) reported that they had helped to interpret the TDM results and recommend vancomycin dose adjustments in >75% of cases. These pharmacists provided recommendations to improve the TDM service (Figure 3), including the need for a more rapid turnaround time for serum level (76.2%), the development of a standardized guideline for dosing adjustment based on TDM results (73.8%), and more collaboration with physicians (11.9%). The pharmacists also suggested other means of support, such as granting pharmacists the right to order a TDM level and the provision of weekend clinical TDM services from pharmacists.

### Targeted Trough Ranges for Specific Types of Infections

**Figure 4:**
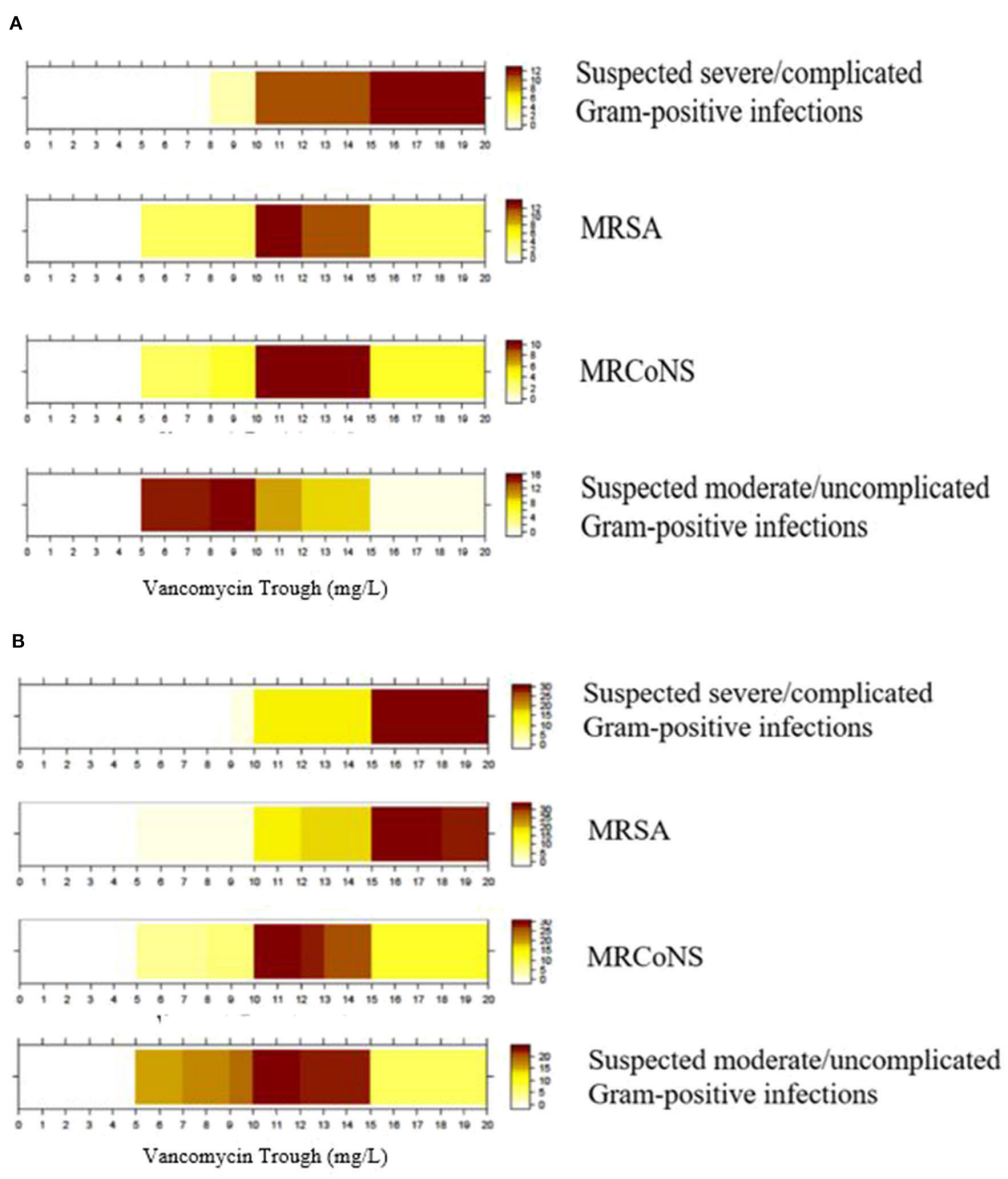
Targeted trough range (mg/L) for vancomycin prescribed for different indications. **(A)** Physicians' target trough range (*n* = 23). **(B)** Pharmacists' target trough range (*n* = 43). Color intensity is directly proportional to the proportion of respondents who selected that though range. MRSA, Methicillin-resistant *Staphylococcus aureus*; MRCoNS, Methicillin-resistant coagulase-negative *Staphylococci*; Examples for complicated infections include bacteraemia, osteomyelitis, meningitis, severe pneumonia, endocarditis, and deep-seated infections.

The color-coded heat graphs revealed that both physicians and pharmacists reported targeted vancomycin trough levels of 15–20 mg/L for a suspected severe/complicated Gram-positive infection when intermittent infusion of vancomycin is administered (Figures 4A,B, respectively). For MRSA infection, physicians and pharmacists reported target trough levels of ~10–15 and 15–20 mg/L, respectively. Both groups of respondents reported targeted trough levels of 10–15 mg/L for confirmed cases of MRCoNS. The respondents' targeted trough levels for suspected moderate/uncomplicated Gram-positive infections ranged between 5 and 15 mg/L. However, physicians tended to prefer a lower trough range of 5–10 mg/L, while pharmacists preferred a range of 10–15 mg/L. Only one physician indicated that their institution adopted an AUC/MIC ≥400 as a therapeutic indicator. Graphical inspection revealed that though responses differ among each respondent, the overall pattern did not differ much across districts for MRSA, MRCoNS, and suspected moderate/uncomplicated Gram-positive infections (Supplementary Material 8).

Our exploratory analysis revealed no statistical difference between the targeted trough ranges reported by physicians and pharmacists for suspected severe/complicated Gram-positive infections (*P* = 1.00), MRSA infections (*P* = 0.49), MRCoNS infections (*P* = 1.00), and suspected moderate/uncomplicated Gram-positive infections (*P* = 0.74).

## Discussion

This study aimed to describe the prescribing preferences of physicians and pharmacists regarding the initial IV intermittent vancomycin dosing and TDM in multiple NICU/PICUs of Hong Kong. Our results demonstrate that these clinicians used varying drug references and vancomycin dosing guidelines in their practices. Generally, the trends in vancomycin target trough levels were similar between the physicians and pharmacists. The highest target trough level was reported for suspected severe/complicated Gram-positive and MRSA infections, followed by MRCoNS and suspected moderate/uncomplicated Gram-positive infections.

Although the AUC/MIC ratio is the primary predictive therapeutic parameter during a vancomycin regimen, the monitoring of trough concentrations as a surrogate parameter is a common practice in clinical settings. Current guidelines regarding vancomycin TDM in adult patients with MRSA infection recommend aggressive dosing when the MIC of the organism is ≤1 mg/L, with a target AUC/MIC ratio >400. This goal is to increase the vancomycin dose exposure and ensure adequate serum and tissue concentrations, particularly in critically ill patients such as those with sepsis, infective endocarditis, meningitis, and pneumonia. Extrapolating adult data to the pediatric population, an expert consensus indicated that an AUC/MIC ratio >400 corresponds to a targeted trough concentration of 15–20 mg/L at a MIC >1 mg/L (22–24). However, our study revealed that the majority of physicians tended to select a target trough level of 10–15 mg/L for MRSA infection, which is lower than the level of 15–20 mg/L suggested by clinical guidelines. This finding has a few implications. First, a clinical consensus on the targeted trough among physicians and pharmacists is needed. Second, with a set of pre-determined trough levels for different clinical scenarios, our future work is to conduct in-house drug utilization studies to evaluate whether the targets are achieved with current dosing strategies. This will shed light on the appropriateness and adequacy of existing dosing approaches. Third, to address concerns about an increase in vancomycin treatment failures, we recommend that clinicians should actively monitor MIC trends within each institution and review the targeted trough ranges. This information should be used to advise the appropriate empirical vancomycin dosing in this special population.

Our results also demonstrated that physicians and pharmacists used different drug references. This practice may lead to a variety of initial dosing regimens. This lack of consensus regarding dosing is illustrated by the wide variations in dosing regimens and targeted therapeutic levels across the reference guidelines (Supplementary Materials 1, 2). Consequently, two studies reported that ~75% of vancomycin dosing regimens designed with respect to the British National Formulary for Children (BNFc) and Neofax recommendations do not achieve the target trough levels (5, 6). Although few studies have involved the pediatric population, the optimization of initial vancomycin dosing to reduce the time required to reach a therapeutic serum level has been proven to improve clinical outcomes. Accordingly, this process should be a goal of therapy (4). However, it is difficult to achieve dose optimization in the neonatal and pediatric population in the absence of an evidence-based empirical dosing recommendation. Our results obtained from a small population of pediatric physicians and pharmacists in Hong Kong suggest a lack of standardization regarding the administration of vancomycin to NICU/PICU patients. As the pediatric critical care service in Hong Kong moves toward centralization at a new pediatric hospital, this issue may become problematic at the early stage when physicians of different institutions adopt varying prescribing preferences. However, we foresee that the gradual development of a standardized dosing guideline for empirical and targeted vancomycin use and a centralized TDM service may potentially improve the overall care for NICU/PICU patients.

The surveyed physicians and pharmacists considered several factors to be important determinants of the initial vancomycin dosing. They gave unanimous responses on crucial determinants such as post-menstrual age, body weight, severity of infection, and renal function. These parameters have also been reported as factors of concern in the literature and references regarding the selection of an initial vancomycin dosing regimen. Among critically ill neonatal and pediatric patients, changes in renal function, fluid resuscitation, and tissue perfusion can influence the clearance of vancomycin. Importantly, the existing references and guidelines differ with respect to covariates (25). Standardized dosing regimens for pediatric patients, which includes neonates, infants, and children, remain controversial because of the high pharmacokinetic variability within this diverse category. Compared with adults, neonates have a higher extracellular fluid volume and a limited but rapidly increasing capacity for renal elimination. Consequently, vancomycin pharmacokinetics evolve significantly as neonates mature (25). The current landscape of multiple dose recommendations pose difficulties on the clinicians to determine dose that can achieve target AUC/MIC goals. This has led institutions to adopt their own measures of developing internal dosing recommendations based on clinical variables collected from their own specific patient population and through stimulation of real-world data (26, 27). Recent studies have also demonstrated the value of using population pharmacokinetic data to personalize empirical vancomycin dosing. Researchers have begun to assess the validity, predictive performance, and generalizability of a published vancomycin pharmacokinetic model retrospectively through a model simulation based on a cohort of neonatal and pediatric patients (28, 29). The existing literature contains promising evidence supporting the use of pharmacokinetic parameters and computational strategies to estimate the extent of drug exposure in this special population. Our proposed future work is to examine the contribution of the identified clinical variables on serum levels using real-world data through drug utilization studies, and using these clinical variables as predictors in developing dosing calculators. We believe that this approach can be extended to establishing pharmacokinetic-guided dosing calculators of antimicrobials in children with other diseases or ethnic origins.

The TDM of medications with narrow therapeutic windows, such as vancomycin, has long been a standard of practice worldwide. Studies investigating the effects of pharmacist-managed TDM services have repeatedly demonstrated improvements in clinical outcomes. The benefits include an improvement in clinical efficacy, as indicated by reductions in the duration of antibiotic use, length of hospital stay, and mortality (4, 17). In a locally conducted pilot study, a pharmacist-managed TDM service exhibited a nearly 50% reduction in the time required to reach target therapeutic levels (30). Despite the purported benefits of TDM, the availability and scope of this clinical service varies among local public hospitals. The respondents in our study identified various obstacles to the optimization of TDM services in their institutions, including logistical barriers such as a slow laboratory turnover time and a lack of autonomy of pharmacists to modify dosing regimens. Based on our observations of local practices, we note that vancomycin dosage adjustments are usually based on incrementally derived dose adjustments, rather than individualized calculations based on pharmacokinetics. This practice suggests a misconception of the principles of TDM and a significant gap in service. The establishment of a well-defined pharmacist-led TDM service and the use of population-based pharmacokinetic modeling principles may resolve challenges such as the determination of a dose for a target serum level.

These study findings should be considered in the context of several limitations. We used a convenience sampling method and obtained a modest response rate. However, efforts were made to disseminate the survey through representatives (e.g., pharmacy managers) at the participating institutions. To highlight, these institutions include hospitals with the largest NICUs and PICUs in Hong Kong and highest usage rates of vancomycin. This approach may have helped to establish the sampling frame and likely strengthened the validity and applicability of the study findings in Hong Kong. It is inevitable that the aggregate responses might be dominated by the major acute hospitals. However, we also purposively distributed the survey to the smaller institutions to ensure that their responses are not omitted due to suboptimal sampling. We also found that the distribution of respondents across the main districts did appear reasonable. The clinicians' practices were self-reported and may not accurately reflect their actual practices in a clinical setting. Therefore, we are currently conducting drug utilization studies to evaluate antimicrobial usage patterns and the impacts on tangible clinical outcomes in the pediatric population. As this study is descriptive in nature, we emphasize that the findings should not be considered the primary and sole evidence on any aspect of this subject. Our study should be interpreted as an effort to understand the perceptions and practices of the pediatric critical care medicine community in Hong Kong. More importantly, findings of this survey will be used to direct future studies within this theme-based research. For example, we found that physicians and pharmacists collectively indicated a target trough of 15–20 mg/L for suspected severe/complicated Gram-positive infection; we will apply this target trough as the threshold when we evaluate clinical outcomes in the subsequent drug utilization studies, as well as whether the current dosing approach can effectively achieve this target trough range.

## Conclusion

In conclusion, we believe that our findings have important implications. From a practice perspective, a standardized dosing guideline is needed to decrease variability and improve local practice. The clinicians' prescribing practices and the effectiveness of using current dosing guidelines to achieve targeted therapeutic trough levels should be evaluated in future drug utilization studies. The multidisciplinary TDM service in Hong Kong can be improved further by addressing logistic barriers and implementing a well-structured, evidence-based service protocol. From a research perspective, the next phase of this work includes conducting a multi-centered drug utilization studies to review real-world data on vancomycin use in the PICU/NICU. The clinical and outcomes data collected will be used to construct pharmacokinetics model for the PICU and NICU cohorts. Future work will also include the performance of comparative studies to demonstrate the effectiveness of a pharmacokinetics-driven dosing regimen vs. standard practice, particularly within the Chinese pediatric population. Globally, drug-related problems (particularly inappropriate dose selection) are common in NICU/PICU, hence predominating problems of suboptimal treatment. We believe that our systematic approach in identifying and addressing therapeutic gaps may potentially be extended to standardize dosing of antimicrobials and other drugs of narrow therapeutic index in the NICU/PICU. The eventual goal is to achieve personalized, pharmacokinetic-guided dosing of drugs in critically ill neonates and pediatric patients.

## Data Availability Statement

The datasets generated for this study are available on request to the corresponding author.

## Ethics Statement

The studies involving human participants were reviewed and approved by The Chinese University of Hong Kong (CUHK) Survey and Behavioral Research Ethics Committee. Written informed consent for participation was not required for this study in accordance with the institutional requirements.

## Author Contributions

TC, JL, TL, CE, and YC: analysis. TC, JL, and YC: drafting the work. All authors: conception or design of the work, acquisition of data, interpretation of data, revising it critically for important intellectual content, and provide approval for publication of the content.

## Conflict of Interest

The authors declare that the research was conducted in the absence of any commercial or financial relationships that could be construed as a potential conflict of interest.
