# Supplementary material for: Vancomycin Prescribing Practices and Therapeutic Drug Monitoring for Critically Ill Neonatal and Pediatric Patients: A Survey of Physicians and Pharmacists in Hong Kong
Source: Front Pediatr. 2020 Nov 30;8:538298. doi: 10.3389/fped.2020.538298 (PMC7734090; doi:10.3389/fped.2020.538298)
Supplement: Supplementary file 1 [file Table_1.docx]

Supplementary Material 1: Vancomycin Initial Dosage Recommendations for Neonates and Pediatrics

| **Neonates** | |  | | | |  |
| --- | --- | --- | --- | --- | --- | --- |
| **References** | | **Corrected gestational age** | | | | **Dosage** |
| BNF for Children | | <29 weeks | | | | 15mg/kg Q24H |
|  |  | 29-35 weeks | | | | 15mg/kg Q12H |
|  |  | >35 weeks | | | | 15mg/kg Q8H |
| **References** | | **Postmenstrual age** | | **Postnatal age** | | **Dosage** |
| Micromedex Neofax | | ≤ 29 weeks | | 0-14 days | | 10 - 15 mg/kg Q18H |
|  |  |  |  | >14 days | | 10 - 15 mg/kg Q12H |
|  |  | 30-36 weeks | | 0-14 days | | 10 -15 mg/kg Q12H |
|  |  |  |  | >14 days | | 10 - 15 mg/kg Q8H |
|  |  | 37-44 weeks | | 0-7 days | | 10 - 15 mg/kg Q12H |
|  |  |  |  | >7 days | | 10 - 15 mg/kg Q8H |
|  |  | ≥45 weeks | | ALL | | 10 - 15 mg/kg Q6H |
| **References** | | **Body weight** | | **Postnatal Age** | | **Dosage** |
| Lexicomp Neonatal | Weight-based dosing | <1.2 kg | | ≤28 days | | 15 mg/kg Q18 - 24H |
|  |  | 1.2 to 2 kg | | <7 days | | 10 - 15 mg/kg Q12 - 18H |
|  |  |  |  | ≥7 days | | 10 - 15 mg/kg Q8 - 12H |
|  |  | >2 kg | | <7 days | | 10 - 15 mg/kg Q8 - 12H |
|  |  |  |  | ≥7 days | | 10 - 15 mg/kg Q6 - 8H |
|  | Renal function-based dosing | Gestational age | | Serum creatinine | | Dosage |
|  |  | ≤28 weeks | | <0.5 mg/dL | | 15 mg/kg Q12H |
|  |  |  |  | 0.5 - 0.7 mg/dL | | 20 mg/kg Q24H |
|  |  |  |  | 0.8 - 1 mg/dL | | 15 mg/kg Q24H |
|  |  |  |  | 1.1 - 1.4 mg/dL | | 10 mg/kg Q24H |
|  |  |  |  | >1.4 mg/dL | | 15 mg/kg Q48H |
|  |  | >28 weeks | | <0.7 mg/dL | | 15 mg/kg Q12H |
|  |  |  |  | 0.7 - 0.9 mg/dL | | 20 mg/kg Q24H |
|  |  |  |  | 1 - 1.2 mg/dL | | 15 mg/kg Q24H |
|  |  |  |  | 1.3 - 1.6 mg/dL | | 10 mg/kg Q24H |
|  |  |  |  | >1.6 mg/dL | | 15 mg/kg Q48H |
| **References** | | **Postnatal age** | | | | **Dosage** |
| Australian Medicines Handbook Children’s Dosing Companion | | Birth (at term) - 1 week | | | | 15mg/kg Q12H |
|  |  | 1 week - 1 month | | | | 15mg/kg Q8H |
| **Pediatrics** | |  | | | |  |
| **References** | | **Age** | **Dosage** | | | |
| BNF for Children | | 1 month – 18 years | 15 mg/kg Q8H | | | |
| Micromedex Paediatrics | | ≥ 29 days | 10 mg/kg Q6H | | | |
|  |  |  | Invasive MRSA: 15 mg/kg Q6H | | | |
| Lexicomp Paediatrics | | > 60 days | Mild to moderate: 40-45 mg/kg/day divided 6-8H | | | |
|  |  |  | Severe: 45-60 mg/kg/day divided 6-8H | | | |
|  |  |  | Life-threatening, invasive MRSA: | | | |
|  |  |  | 3 months - <2 years | | 70 mg/kg/day divided 6-8H | |
|  |  |  | 2 - <12 years | | 60 mg/kg/day divided 6-8H (if sCr<0.45 mg/dL or >30% MRSA MIC ≥1.5 mcg/mL: 70 mg/kg/day divided 6-8H) | |
|  |  |  | ≥12 years | | 60 mg/kg/day divided 6-8H | |
| Frank Shann | | N/A | 25mg/kg IV then 15-20 mg/kg Q8-12H | | | |
|  |  |  | Severe: 30mg/kg IV then 15-20 mg/kg Q8-12H | | | |
| Infectious Diseases Society of America | | N/A | Severe, invasive: 15 mg/kg Q6H | | | |
| Australian Medicines Handbook Children’s Dosing Companion | | 1 month – 18 years | 15 mg/kg Q6H or 20 mg/kg Q8H or 30 mg/kg Q12H | | | |
