# Supplementary material for: Vancomycin Prescribing Practices and Therapeutic Drug Monitoring for Critically Ill Neonatal and Pediatric Patients: A Survey of Physicians and Pharmacists in Hong Kong
Source: Front Pediatr. 2020 Nov 30;8:538298. doi: 10.3389/fped.2020.538298 (PMC7734090; doi:10.3389/fped.2020.538298)
Supplement: Supplementary file 2 [file Table_2.docx]

Supplementary Material 2: Targeted Trough Levels of Vancomycin in Neonatal and Pediatric Population

| **References** | **Targeted trough level** |
| --- | --- |
| BNF for Children | 10 – 15 mcg/mL |
|  | Less sensitive strains of MRSA: 15 – 20 mcg/mL |
| Micromedex Neofax | 7 – 15 mcg/mL |
| Micromedex Paediatrics | Non-MRSA: 10 – 15 mcg/mL |
|  | MRSA bacteremia, infective endocarditis, osteomyelitis, meningitis, pneumonia, complicated skin and soft-tissue infections, or bone/joint infections: 15 – 20 mcg/mL |
| Lexicomp Neonatal | ~10 mcg/mL |
|  | MRCoNS or enterococcal infection: 5 - 10 mcg/mL |
| Lexicomp Paediatrics | 10 – 15 mcg/mL |
|  | Complicated infection, MIC ≥1 mcg/mL: 15 – 20 mcg/mL |
| Frank Shann | 10 – 15 mcg/mL |
|  | Severe infection: 15 – 20 mcg/mL |
| Infectious Diseases Society of America | Severe, invasive infection: 15 – 20 mcg/mL |
| Australian Medicines Handbook Children’s Dosing Companion | 10 – 15 mg/L (7 – 10 mg/L may be adequate in treating sensitive organisms using 6- or 8- hourly regimens) |
|  | Serious infections: 15 – 20 mg/L |
