# Supplementary material for: Vancomycin Prescribing Practices and Therapeutic Drug Monitoring for Critically Ill Neonatal and Pediatric Patients: A Survey of Physicians and Pharmacists in Hong Kong
Source: Front Pediatr. 2020 Nov 30;8:538298. doi: 10.3389/fped.2020.538298 (PMC7734090; doi:10.3389/fped.2020.538298)
Supplement: Supplementary file 3 [file Image_3.pdf]

Supplementary Material 3: Mixed-methods Approach to Address Appropriate Antimicrobial Use in the Pediatric Population

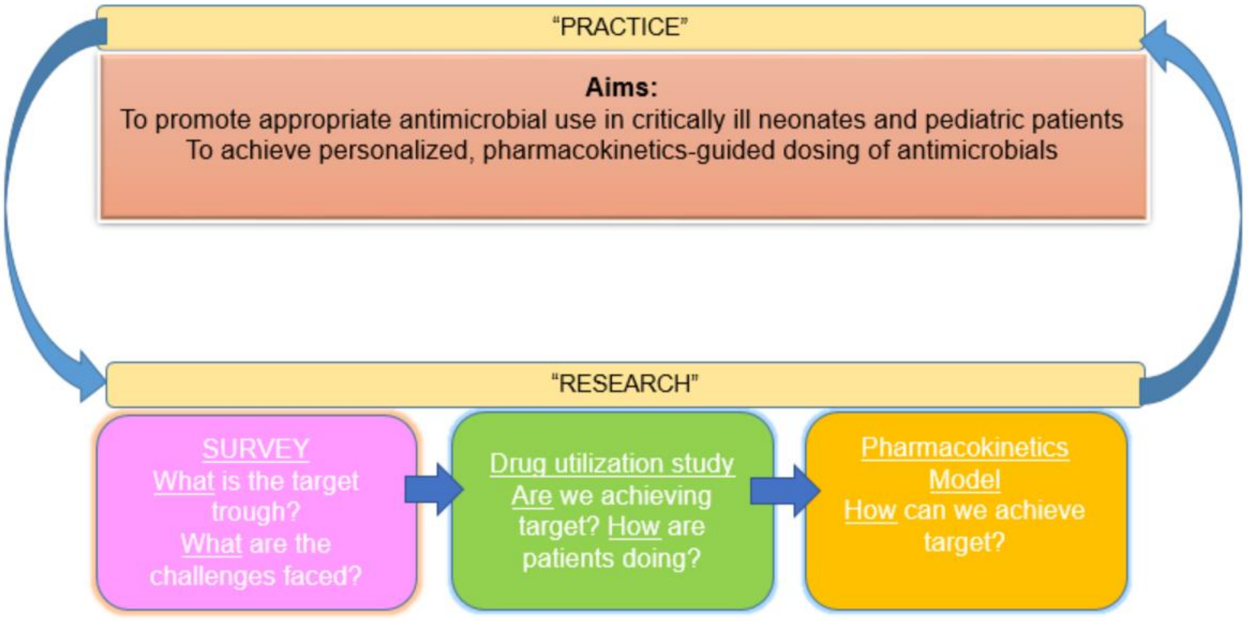

In 2019, a multidisciplinary team consisting neonatologist, pediatricians, and clinical pharmacists initiated a mixed-methods approach to address appropriate antimicrobial use in the pediatric population of Hong Kong. The overarching aim is to implement personalized, pharmacokinetic-guided dosing of antimicrobials in critically ill patients in neonatal and pediatric intensive care units. The first step is to conduct a descriptive survey to gather expert opinions on initial vancomycin dose selection and prescribing behavior. The second step is to review real-world data on vancomycin use through a multi-centered drug utilization study. The third step is to construct a pharmacokinetics model using the clinical data from the drug utilization study and develop a pharmacokinetic-guided dosing calculator. The final step will be to perform comparative studies to demonstrate the effectiveness of a pharmacokinetics-driven dosing regimen versus standard practice.
