# Supplementary material for: Vancomycin Prescribing Practices and Therapeutic Drug Monitoring for Critically Ill Neonatal and Pediatric Patients: A Survey of Physicians and Pharmacists in Hong Kong
Source: Front Pediatr. 2020 Nov 30;8:538298. doi: 10.3389/fped.2020.538298 (PMC7734090; doi:10.3389/fped.2020.538298)
Supplement: Supplementary file 4 [file Table_4.docx]

Supplementary Material 4: Participating Medical Institutions

| **Participating hospitals** | **District^** |
| --- | --- |
| Alice Ho Miu Ling Nethersole Hospital | North Territories |
| Prince of Wales Hospital* | North Territories |
| Tuen Mun Hospital | North Territories |
| Hong Kong Children’s Hospital* | Kowloon Central |
| Kwong Wah Hospital | Kowloon Central |
| Queen Elizabeth Hospital* | Kowloon Central |
| Princess Margaret Hospital | Kowloon West |
| Yan Chai Hospital | Kowloon West |
| United Christian Hospital | Kowloon East |
| Pamela Youde Nethersole Eastern Hospital | Hong Kong Island |
| The Duchess of Kent Children's Hospital at Sandy Bay | Hong Kong Island |
| Queen Mary Hospital* | Hong Kong Island |

^Hong Kong is geographically divided into three main districts: North Territories, Kowloon (Central and East/West), and Hong Kong Island.

*Major acute hospitals or hospitals that provides specialized paediatric services, spread across the three main districts.
