# Supplementary material for: Vancomycin Prescribing Practices and Therapeutic Drug Monitoring for Critically Ill Neonatal and Pediatric Patients: A Survey of Physicians and Pharmacists in Hong Kong
Source: Front Pediatr. 2020 Nov 30;8:538298. doi: 10.3389/fped.2020.538298 (PMC7734090; doi:10.3389/fped.2020.538298)
Supplement: Supplementary file 5 [file Table_5.docx]

Supplementary Material 5: Respondents stratified by Districts

| District^ | No of respondents |
| --- | --- |
| A | 11 |
| B | 28 |
| C | 11 |
| D | 16 |

^Districts are de-identified to ensure anonymity of the respondents and their respective institutions. The same de-identifiers are used to present trough level results by each respondent, stratified by district, in Supplement 8.
