# Supplementary material for: Vancomycin Prescribing Practices and Therapeutic Drug Monitoring for Critically Ill Neonatal and Pediatric Patients: A Survey of Physicians and Pharmacists in Hong Kong
Source: Front Pediatr. 2020 Nov 30;8:538298. doi: 10.3389/fped.2020.538298 (PMC7734090; doi:10.3389/fped.2020.538298)
Supplement: Supplementary file 6 [file Table_6.docx]

Supplementary Material 6: Dosing Guidelines and References Used by Respondents

|  | NICU | | | PICU | | |
| --- | --- | --- | --- | --- | --- | --- |
| References | **Physicians**  n (%) | **Pharmacists**  n (%) | **Total**  n (%) | **Physicians**  n (%) | **Pharmacists**  n (%) | **Total**  n (%) |
| UpToDate^®^/Lexicomp^®^ Paediatric and Neonatal | 3 (13.0) | 22 (51.2) | 25 (37.9) | 7 (30.4) | 32 (74.4) | 39 (59.1) |
| Frank Shann Drug Doses | 3 (13.0) | 0 | 3 (4.5) | 17 (73.9) | 5 (11.6) | 22 (33.3) |
| Micromedex^®^ Neofax and Paediatric Essentials | 11 (47.8) | 28 (65.1) | 39 (59.1) | 3 (8.7) | 13 (`30.2) | 15 (22.7) |
| BNF (British National Formulary) for Children | 5 (21.7) | 12 (27.9) | 17 (25.8) | 9 (39.1) | 17 (39.5) | 26 (39.4) |
| Local hospital guidelines | 11 (47.8) | 11 (25.6) | 22 (33.3) | 4 (17.4) | 7 (16.3) | 11 (16.7) |
| Disease-based guidelines^#^ | 1 (4.3) | 0 | 1 (1.5) | 2 (8.7) | 1 (2.3) | 3 (4.5) |
| My own clinical judgement | 0 | 1 (4.3) | 1 (1.5) | 0 | 1 (2.3) | 1 (1.5) |
| Others* | 0 | 0 | 0 | 0 | 2 (4.7) | 2 (3.0) |

# Diseases-based guidelines include the Infectious Diseases Society of America (IDSA) Guideline

*Others include guidelines and formulary developed by SickKids and Great Ormond Street Hospital
