# Supplementary material for: Vancomycin Prescribing Practices and Therapeutic Drug Monitoring for Critically Ill Neonatal and Pediatric Patients: A Survey of Physicians and Pharmacists in Hong Kong
Source: Front Pediatr. 2020 Nov 30;8:538298. doi: 10.3389/fped.2020.538298 (PMC7734090; doi:10.3389/fped.2020.538298)
Supplement: Supplementary file 7 [file Table_7.docx]

Supplement 7: Reasons for not Adjusting Vancomycin Dose despite Suboptimal Serum Levels (n=66)

| Reasons | Respondents (%) |
| --- | --- |
| For empiric use pending culture results | 12 (18.2) |
| For short-term use (<72 hours) | 38 (57.6) |
| For surgical prophylaxis | 43 (65.2) |
| Prescribed dose always achieves target levels | 0 |
| Patient has good or normal renal function | 5 (7.6) |
| Time lag for serum tests to be available | 2 (3.0) |
| Target levels are unclear for unknown pathogen | 1 (1.5) |
| Serum levels are not useful in patient management | 1 (1.5) |
| Patient is hemodynamically unstable | 2 (3.0) |
| TDM requires too much blood drawing | 2 (3.0) |
| The patient does not have a severe infection | 0 |
