# Supplementary material for: Vancomycin Prescribing Practices and Therapeutic Drug Monitoring for Critically Ill Neonatal and Pediatric Patients: A Survey of Physicians and Pharmacists in Hong Kong
Source: Front Pediatr. 2020 Nov 30;8:538298. doi: 10.3389/fped.2020.538298 (PMC7734090; doi:10.3389/fped.2020.538298)
Supplement: Supplementary file 8 [file Image_8.pdf]

Supplementary Material 8: Trough Levels by each Respondent and Stratified by District

A: Suspected severe/complicated Gram positive infections

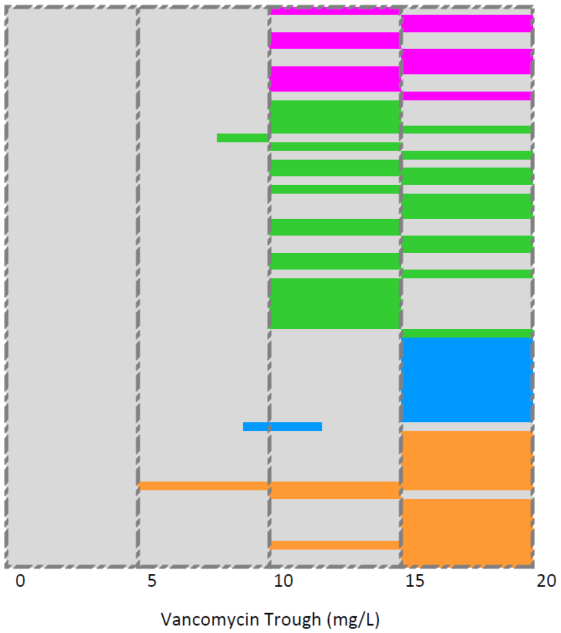


B: Methicillin-resistant Staphylococcus aureus

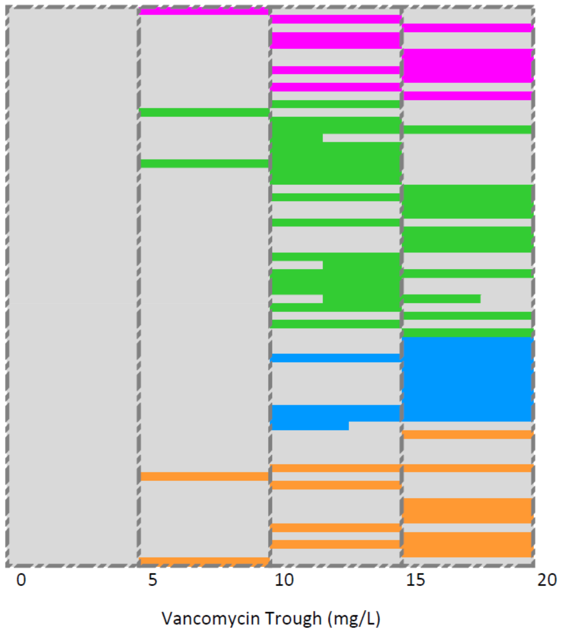


C: Methicillin-resistant coagulase-negative staphylococci

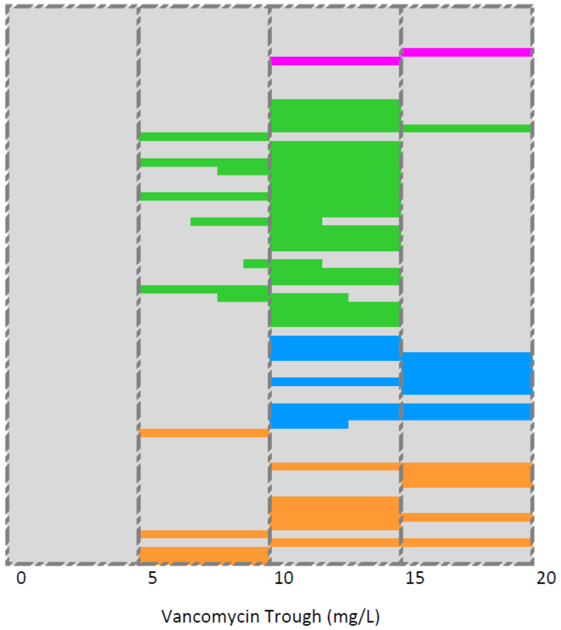


D: Suspected moderate/ uncomplicated Gram positive infections

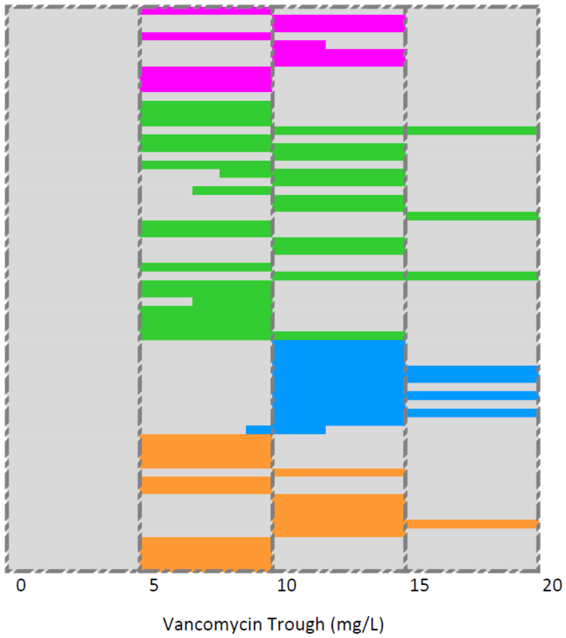


Each bar represents one respondent. Each color represents one district.
